# Eyelid reconstruction with radial free forearm flap: functional and esthetic outcomes

**DOI:** 10.1093/jscr/rjaf688

**Published:** 2025-09-06

**Authors:** Frank A Álvarez Vásquez, Marco A Paipilla, Felipe Ángel Rodríguez, Mauricio A Uribe Rodríguez, Iván E Rodríguez Mantilla

**Affiliations:** Faculty of Medicine, Universidad del Rosario, Bogotá, Colombia; Faculty of Medicine, Universidad El Bosque, Bogotá, Colombia; Faculty of Medicine, Universidad de La Sabana, Chía, Colombia; Faculty of Medicine, Universidad El Bosque, Bogotá, Colombia; Department of Plastic Surgery, Fundación Universitaria de Ciencias de la Salud, Bogotá, Colombia

**Keywords:** radial forearm flap, eyelid reconstruction, reconstructive surgery, periorbital defects, microvascular flap, facial reconstruction

## Abstract

Periorbital defects resulting from oncologic resections, trauma, or congenital malformations pose a complex reconstructive challenge, due to the need to simultaneously restore eyelid function and facial esthetics. We present the case of a male patient in his seventh decade of life with a right orbitomalar squamous cell carcinoma, who underwent a wide oncologic resection involving the upper and lower eyelids, as well as the malar and infraorbital regions. Reconstruction was performed using a radial free forearm flap from the left arm, including the palmaris longus tendon, which was strategically anchored to the medial canthus and orbital rim to provide dynamic eyelid support. This technique allowed for satisfactory anatomical and functional restoration. The microvascular radial forearm flap stands out for its thinness, pliability, and reliable vascularity, making it a suitable alternative to bulkier flaps such as the anterolateral thigh flap, particularly in the reconstruction of extensive and anatomically complex defects.

## Introduction

Soft tissue defects of the eyelid region secondary to oncologic resections, congenital malformations, or severe trauma represent a significant challenge in plastic and ophthalmic surgery due to the complex anatomy and the critical role of eyelid closure [1, 2]. These defects may compromise vital structures, affecting ocular protection and proper lacrimal drainage [3].

The choice of reconstructive method depends on several factors, including the size and depth of the defect, availability of adjacent tissue, patient age, comorbidities, presence or absence of radiodermatitis, and the surgeon's expertise [2, 4]. The primary goal is to fully restore eyelid function while achieving an optimal esthetic outcome and minimizing functional sequelae. Various techniques have been described for lower eyelid reconstruction, including local flaps such as the cheek advancement and rotation flap (Mustardé), the semicircular Tenzel flap, the upper eyelid tarsoconjunctival flap (Hughes), and the Cutler-Beard flap [2, 4, 5]. However, in cases where local options are insufficient or contraindicated, free microvascular flaps—such as the radial free forearm flap (RFFF) —have proven to be a versatile and reliable alternative for restoring cutaneous and mucosal coverage as well as tendinous support [1, 2].

In this context, we present a case report documenting the use of a RFFF including the palmaris longus tendon for lower eyelid reconstruction. Preoperative considerations, surgical technique, and postoperative management are discussed, highlighting the advantages and satisfactory outcomes achieved with this reconstructive approach.

## Case report

**Figure 1:**
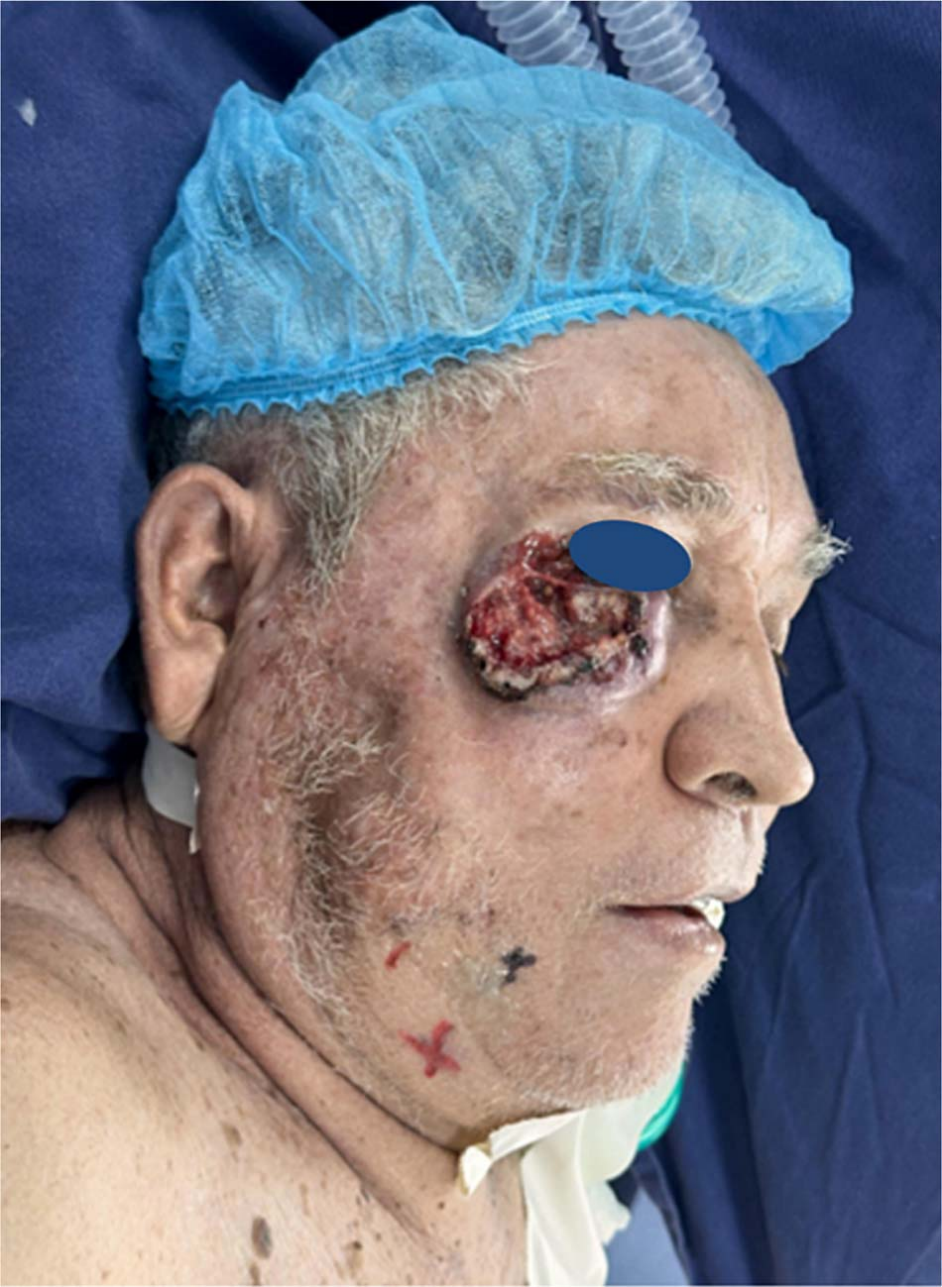
Ulcerated and infiltrative lesion in the right orbitomalar region, with complete destruction of the lower eyelid and extension to adjacent cheek tissues.

A male patient in his seventh decade of life, with a medical history of insulin-dependent type 2 diabetes mellitus and benign prostatic hyperplasia, was referred from the dermatologic oncology department due to an exophytic, ulcerated, and infiltrative lesion in the right orbitomalar region, ⁓7 × 6 cm in size, extending to the ipsilateral cheek (Fig. 1). Histopathological analysis confirmed a well-differentiated keratinizing large cell squamous cell carcinoma, clinically staged as T3N0M0.

**Figure 2:**
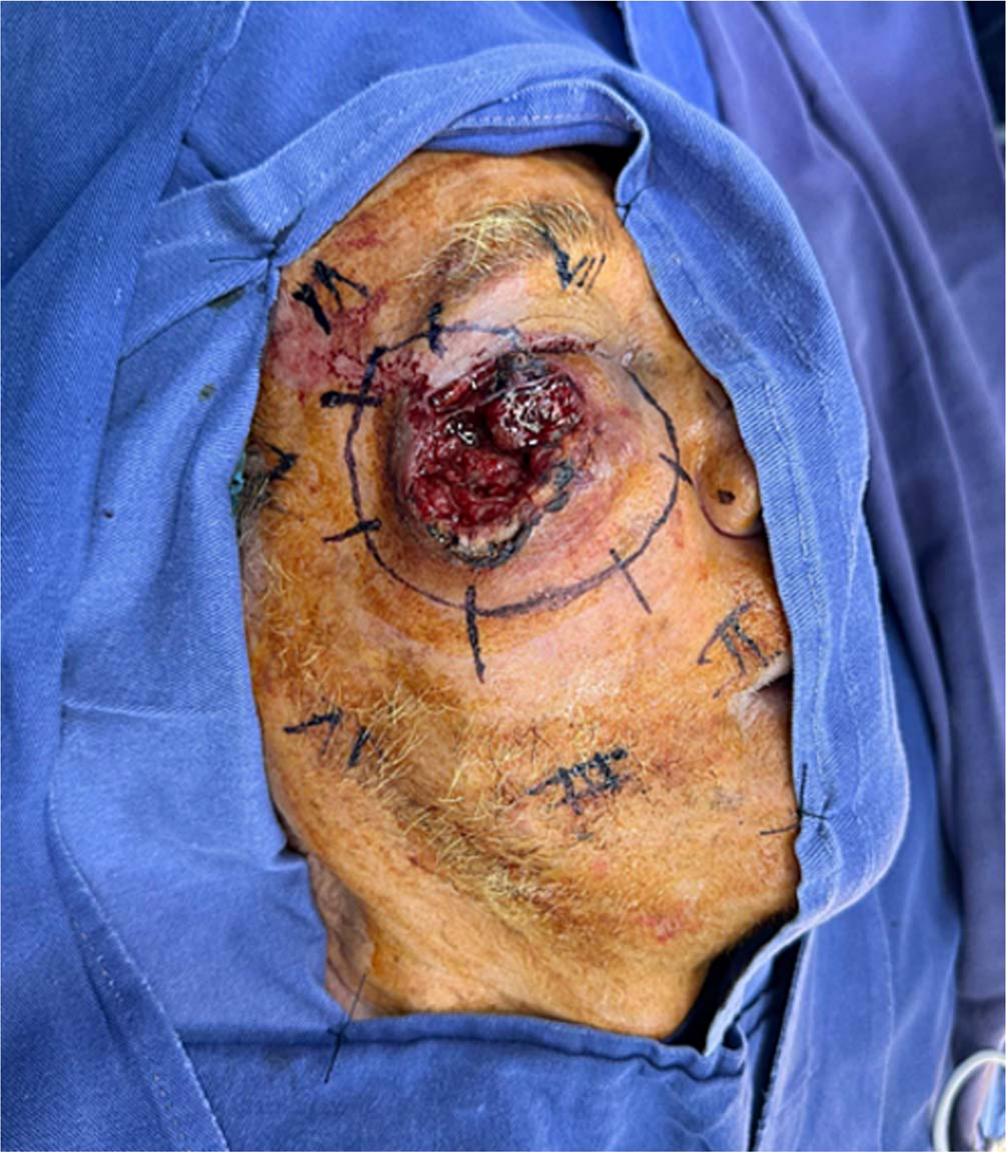
Intraoperative surgical marking showing 1 cm oncologic margins around the lesion.

**Figure 3:**
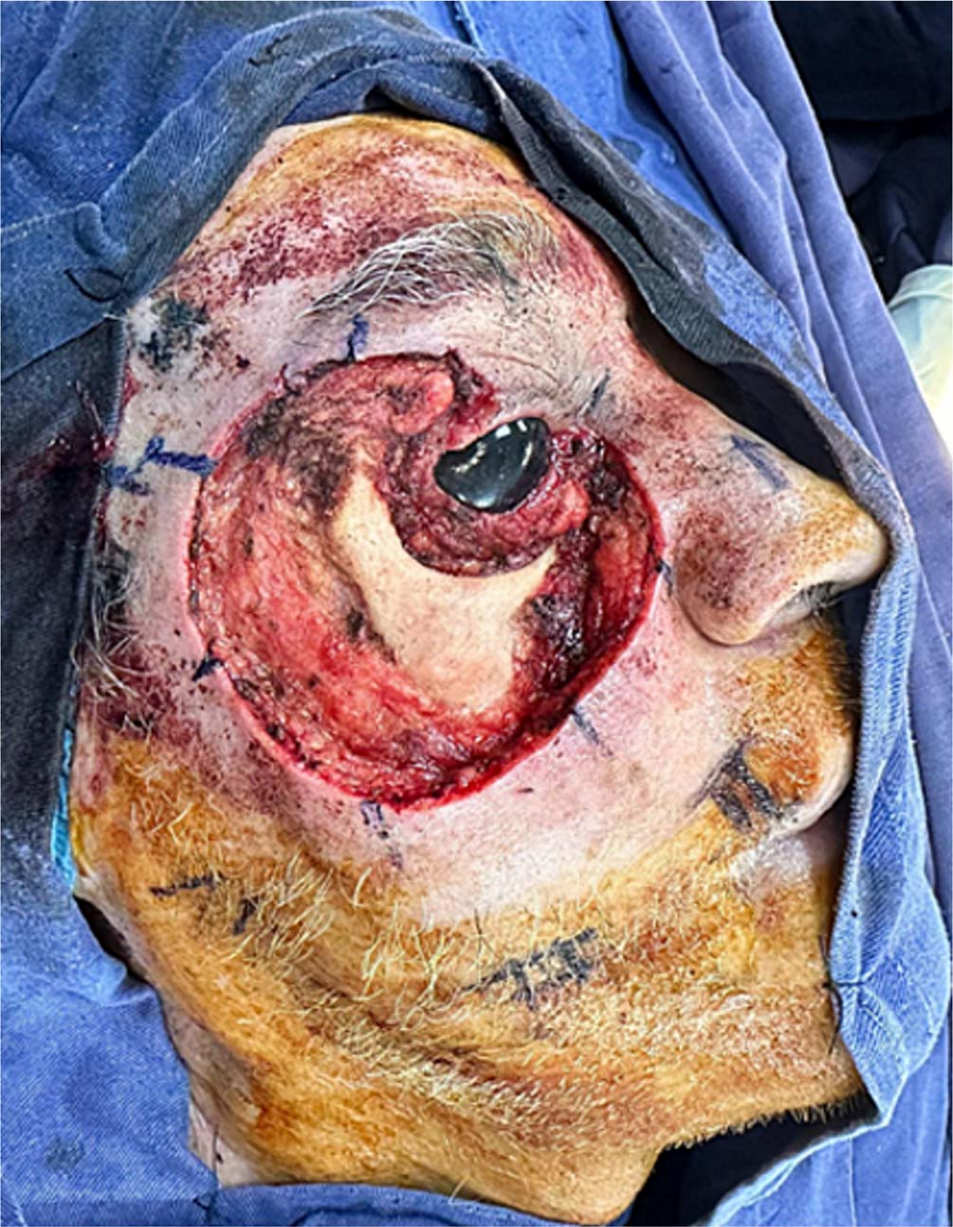
Extensive and complex defect secondary to tumor resection with oncologic margins, involving multiple anatomical subunits including the upper eyelid, lower eyelid, malar, and infraorbital regions.

Physical examination revealed an ulceroinfiltrative lesion in the right orbitomalar region, with complete destruction of the lower eyelid, involvement of the lateral canthus, and close contact with the orbital floor, although without macroscopic evidence of bony invasion. Computed tomography imaging showed no lymph node metastases or extension to the parotid gland or bony structures. The patient underwent surgery performed by the head and neck team, involving wide oncologic resection of the orbitomalar lesion with 1 cm margins and orbital decompression (Fig. 2). This resulted in a complex soft tissue defect on the right hemiface, involving multiple facial subunits: the upper eyelid (anterior lamella), lower eyelid (both anterior and posterior lamellae), malar region, and infraorbital area (Fig. 3).

**Figure 4:**
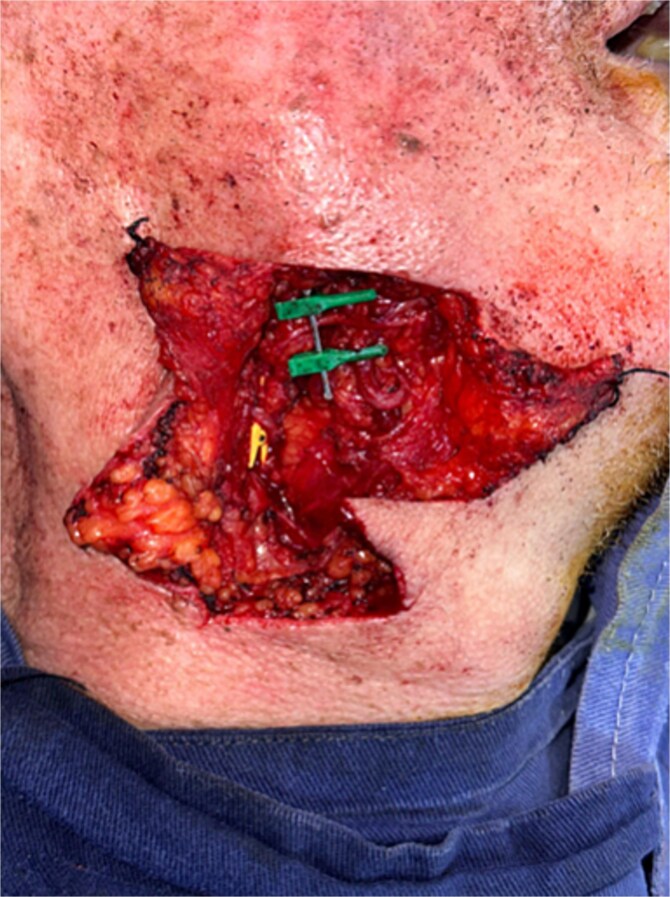
Dissection of vessels in the right submandibular region identifying the facial artery and external jugular vein for microvascular anastomosis.

**Figure 5:**
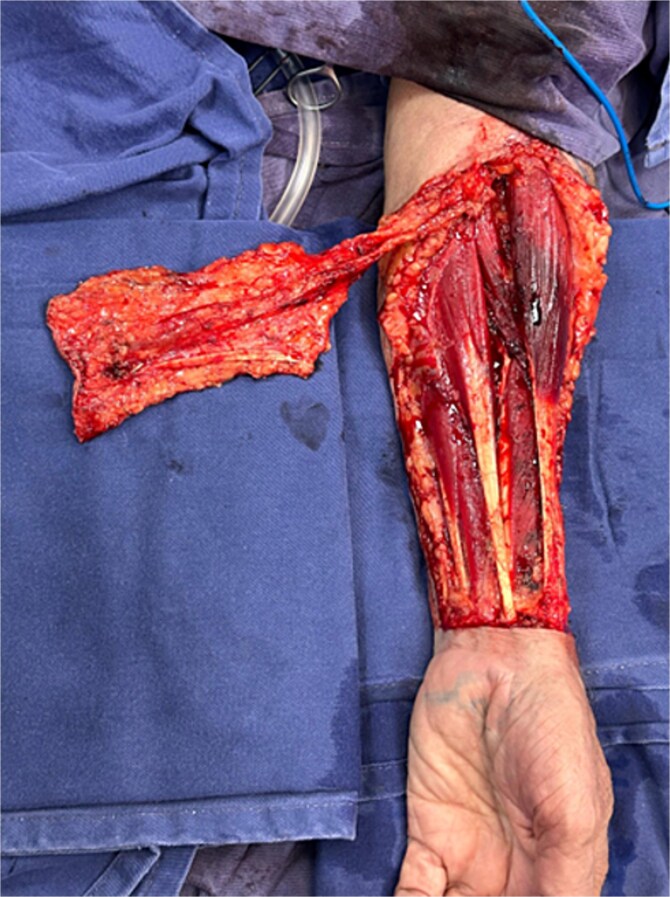
Design and elevation of the free radial forearm flap from the left forearm. Inclusion of the palmaris longus tendon and the cephalic venous system is observed.

Given the size and complexity of the defect, reconstruction with a RFFF was chosen. Under microscopic magnification and using microsurgical instruments, a Z-shaped incision was made in the right submandibular region to access and prepare the recipient vessels, identifying the facial artery and external jugular vein (Fig. 4). In the left forearm, dissection of the radial vascular bundle was performed, including the cephalic vein and the palmaris longus tendon—the latter used for eyelid suspension. A composite radial forearm flap was harvested, incorporating skin, the cephalic venous system, and the tendon (Fig. 5).

**Figure 6:**
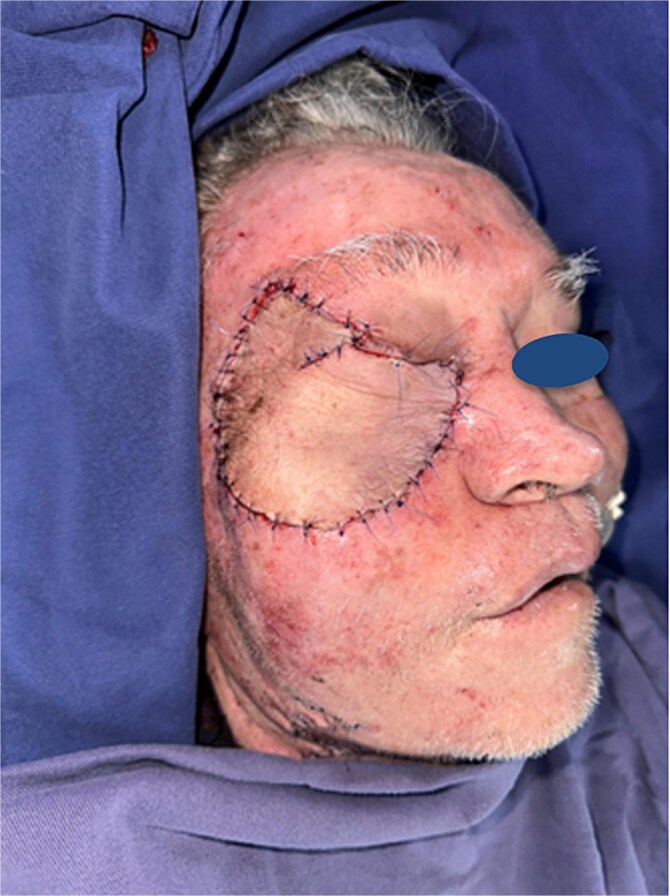
Complete defect coverage following transfer of the radial forearm flap.

The flap was then transferred to the right hemiface. End-to-end microvascular anastomosis was performed between the flap’s radial artery and the recipient facial artery. The flap was tunneled subcutaneously to the cheek region for positioning. The palmaris longus tendon was anchored to the medial canthus and the orbital rim, providing dynamic eyelid suspension. Layered closure was completed, achieving full coverage of the defect (Fig. 6).

A temporary tarsorrhaphy was performed for ocular protection, and the use of scleral contact lenses was recommended in the immediate postoperative period. Due to the residual donor site defect on the volar aspect of the left forearm, split-thickness skin grafts harvested from the left thigh were used for coverage.

**Figure 7:**
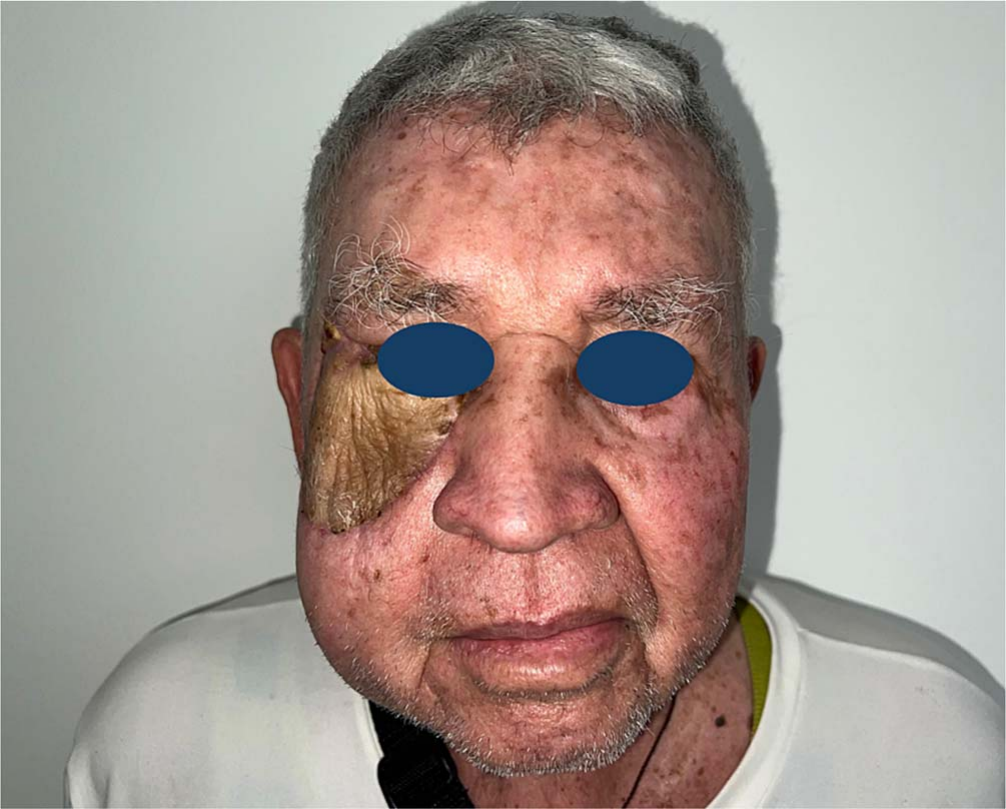
Postoperative follow-up: Satisfactory functional and esthetic outcome, viable flap with no evidence of complications or tumor recurrence.

During outpatient follow-up, the flap remained viable, with no signs of arterial or venous compromise, dehiscence, or local complications. There was no evidence of locoregional recurrence, and the functional integrity of the reconstructed eyelid was preserved without ectropion. Additionally, no visual acuity impairments were observed. The patient showed satisfactory clinical evolution with favorable functional and esthetic outcomes (Fig. 7).

## Discussion

The RFFF also known as the "Chinese flap," was first described in 1978 at the Shenyang Military Hospital (China) by Drs. Yang Guofan and Gao Yuzhi. It is a fasciocutaneous flap based on the radial artery [2, 3]. Initially employed for intraoral and pharyngeal reconstruction, it has since become a well-established and reliable option in reconstructive microsurgery due to its ability to provide both cutaneous coverage and structural support [6–8].

This flap is characterized by its thinness, excellent skin quality, and reliable vascularity. It allows for the inclusion of fascial, tendinous, and even neural components, and its sufficiently long vascular pedicle enables secure anastomosis to distant recipient vessels [1–3, 9]. These features contribute to stable coverage with favorable esthetic and functional outcomes [10–12].

A key advantage is the possibility of incorporating the palmaris longus tendon, present in ⁓85% of the population [4]. This component is especially valuable in eyelid reconstruction, as it can be used for canthal suspension or as a structural replacement for the tarsal ligament. It reduces the risk of ectropion and ensures proper eyelid function by allowing the tendon to be sutured to the remnant of the levator muscle or its aponeurosis, or alternatively to the periosteum of the orbital rim, thereby restoring the necessary mechanical and dynamic action for effective eyelid elevation [2, 3, 5].

Despite its advantages, the RFFF presents certain drawbacks, including the sacrifice of the radial artery and notable donor-site morbidity. This often results in a visible scar on the anterolateral and volar forearm, and typically requires coverage with a split-thickness skin graft [13, 14]. Additional risks include restricted hand mobility, poor graft take, or exposure of the flexor tendons due to partial or complete graft failure [14, 15]. Moreover, this procedure must be performed by a team experienced in microsurgery, given the technical precision and expertise required to ensure flap viability and optimal functional outcomes [16].

In the case presented, inclusion of the palmaris longus tendon allowed for effective suspension of the lower eyelid, anchoring it to the medial canthus and orbital rim. This maneuver was essential in preventing complications such as ectropion, corneal exposure, and keratitis. Postoperative follow-up confirmed complete flap viability, with no signs of tissue compromise, dehiscence, or local recurrence, and with preservation of visual acuity. These results underscore the efficacy of this technique in achieving both functional and esthetic reconstruction.

## Conclusion

The reconstruction of extensive and complex defects in the eyelid region poses a significant challenge. In clinical and surgical scenarios where local and regional options are insufficient, the RFFF emerges as a robust, reliable, and versatile solution, capable of providing thin, well-vascularized tissue coverage—ideal for restoring both function and esthetics. Notably, its ability to offer dynamic structural support through tendon fixation to orbital structures helps prevent complications such as ectropion and ensures adequate protection of the globe, resulting in favorable functional and esthetic outcomes.
